# The Problem of Venereal Disease

**Published:** 1920-01-17

**Authors:** 


### The Problem of Venereal Disease.

A good deal of confusion has arisen in the public mind with regard to the distinction between the National Council for Combating Venereal Diseases and the recently-formed Society for the Prevention of Venereal Disease. Both bodies take their stand on common ground in desiring the reduction and final elimination of the terrible scourge of syphilis and gonorrhœa from our national life. But they approach and deal with the problem from entirely different points of view.

Basing their opinion on the findings of the Royal Commission on Venereal Disease and the experience gained during the war, the National Council contends that the strongest weapons available are, first, the education of all classes and sections of the community as to the causes, nature, and results of those diseases, and, secondly, the earliest possible treatment of infected persons. They also recognise that immediate local cleanliness after exposure to the risk of infection will reduce that risk, but, unfortunately, it is by no means an infallible preventive. Where propaganda stating that it is so has taken place, the amount of venereal disease has materially increased—*vide* the incidence among the Australian troops, starting as it did at 130 per thousand and rising to 147 per thousand, as compared with the British troops' incidence of thirty-eight per thousand.

The Society for the Prevention of Venereal Disease consider that immediate self-disinfection is the only measure worthy of consideration from either a medical or social point of view. In this they are in disagreement with the opinion expressed by Lord Sandhurst on behalf of the Government during the debate in the House of Lords on December 10, when he stated that "even in circumstances specially favourable to instruction in the method proposed the risk of venereal disease is by no means certainly avoided. . . . How much more, then, would this be the case, and how far removed should we still be from secured immunity, with the civil population, in respect of whom the arrangements as to instruction and discipline adopted for the military would obviously be quite impossible?" The same view is held by an almost unanimous majority of medical officers of health and clinic officers throughout the country. Many of those who support the policy of the Society for the Prevention of Venereal Disease bear illustrious names rightly honoured in the medical world—but they do not represent those medical men and women who have had long and intimate experience of the practical problems involved in the successful prevention and treatment of venereal disease. The Society for the Prevention of Venereal Disease is therefore completely at variance with the National Council in regarding immediate self-disinfection as the one and only practical measure by which venereal disease can be exterminated. The National Council considers it but one of several weapons to be added to the armoury of education and prompt treatment.

